# No-Reflow Phenomenon and Endothelial Glycocalyx of Microcirculation

**DOI:** 10.1155/2012/859231

**Published:** 2011-11-29

**Authors:** Alexander V. Maksimenko, Askar D. Turashev

**Affiliations:** Institute of Experimental Cardiology, Russian Cardiology Research-and-Production Complex, 3rd Cherepkovskaya Street 15A, Moscow 121552, Russia

## Abstract

The progress in reperfusion therapy dictated the necessity for developing new tools and procedures for adjacent/additional therapy of acute cardiovascular disorders. The adjacent therapy is targeted on the damage of the microcirculation, leading to the unfavorable prognosis for the patients. The no-reflow phenomenon holds special place in the multifactorial etiology of the microcirculation disorders, offering a new challenge in treating the patients associated with ST-segment elevation on ECG at myocardial infarction. One of the numerous causes of no-reflow, the influence of the endothelial glycocalyx of the microcirculation, is analyzed. The results obtained in the studies of the endothelial glycocalyx ultrastructure are generalized, the effect that the fragments of the glycocalyx glycosaminoglycans have on the function of the vascular wall is demonstrated. The trends in searching for correlations between the thickness of the capillary glycocalyx and the cardiovascular disease risk are noted.

## 1. Introduction

The continuous improvement of the methods, tools, and medications for reperfusion therapy is motivated by the necessity to reduce the high morbidity and mortality rates for cardiovascular disorders. Acute coronary syndrome is a critical manifestation of ischemic heart disease. The onset of this syndrome can lead to unstable angina pectoris attacks, to the acute myocardial infarction (AMI), and to the sudden cardiac death. The size of myocardial infarction serves as the primary determinant of the prognosis for such patients. The mortality rate over the first six months in patients with infarct size over 20% left ventricle was 2%, and increased to 4.5%, when the lesion size was higher than 35% [1]. An understandable significance of reduction in AMI size, however, did not find a reliable clinical confirmation in the studies [2–4]. The reason is that the reduction of AMI size (although it has a significant effect on the prognosis in the patients with the lesion size of 20% left ventricle and more) is achieved more often in the patients who have smaller lesions (<20% left ventricle), that is, in the patients who already have a favorable prognosis [5]. Therefore, a successful treatment of severe cases is of especial importance for reducing mortality rates in the patients with AMI. The contemporary reperfusion therapy limits AMI sizes approximately by 50% of ischemized area in half of the patients with AMI, leaving out a quarter of the patients with more than 75% of the lesion in the risk zone. This sets an objective of reducing the infarct size from 75% of the lesion or higher down to less than 40% [5]. In a quarter of the patients with AMI this goal could not be accomplished by using the required reperfusion therapy alone, and so an adjunctive (additional) therapy is necessary [6]; the purpose of this adjacent (adjunctive) therapy is to reduce the infarction damage of the left ventricle down to 20% or less [5].

## 2. Necessity for Adjacent Therapy in Treating Disorders of Microcirculation/Tissue Perfusion

One of the reasons for severe progression of AMI is the damage of the tissue perfusion/microcirculation [7]. The positron emission tomography shows that some of the patients with AMI have reduced tissue perfusion caused by the damage of the capillaries induced by ischemia and reperfusion, even after the successful thrombolysis in the infarct-related artery (patency rates reaching TIMI grade 3). As a result, the function of the left ventricle is not restored, which leads to the unfavorable prognosis for these patients. The functional recovery of the affected myocardium was observed only when the patency of the obstructed vessel and an adequate restoration of tissue permeability were ensured [7]. An adjunctive therapy is required for the functional restoration of the microvasculature [6]. The necessity of additional therapeutic intervention for these disorders in the case of complicated AMI was demonstrated by using echocardiography, nuclear magnetic resonance, Doppler scanning [8], angiography (employing semiquantitative classification of microcirculatory perfusion levels) [9], electrocardiography, by measuring plasma levels of the myocardial proteins (creatine kinase MB, troponin T or I, myoglobin) [10, 11], and using positron emission tomography [12]. These data helped to move from the concept of open artery to the achievement of the “optimal reperfusion,” designed, aside from the reperfusion of an occluded artery, to eliminate the dysfunction of microcirculation, improve tissue perfusion, and reduce the “reperfusion” damage [13]. The current situation shows that the use of adjunctive therapy in patients with acute cardiovascular disorders may have various purposes [6, 13]. This review is primarily focused on the problems associated with disorders of microcirculation.

## 3. Disturbances of Microcirculation. No-Reflow Phenomenon

Microcirculation is a part of blood circulation system. Oxygen, nutrients, hormones, and metabolites are exchanged between the circulating blood and the parenchymal cells in the microcirculation bed. The microvessels are divided by their anatomic characters and the direction of blood flow into arterioles, capillaries, and venules, the diameter of which is thought to be less than 100 [14] or 200 *μ*m [15]. Arterioles, the thin branches of arteries, are the principal resistance component of microcirculation. The venules serve as a large reservoir of low pressure (capable to hold up to 75% total blood volume), through which the blood returns into the heart. The main function of capillaries is to facilitate the exchange of molecules between blood and tissues. An adequate capillary circulation is a necessary prerequisite for normal perfusion and organ functioning. Capillary patency is the principal determinant of capillary tissue perfusion and is characterized by the functional capillary density (FCD). The latter is defined as the number of functioning capillaries (i.e., the capillaries with circulating erythrocytes) per tissue area. The major distinctive feature of microcirculation is its heterogeneity (in terms of blood flow distribution, FCD, expression of vasoactive products, etc.). The disorders of the microcirculation are sepsis, hypovolemic and cardiogenic shock, and no-reflow phenomenon (the failure to restore the microflow) [14, 16]. It is believed that in the case of sepsis (associated with local hypoxia and organ dysfunction) and cardiogenic shock (i.e., a response to the generalized inflammation with compensatory redistribution of blood volume/due to active venoconstriction/with reduction of the peripheral vascular capacity), the disturbances of microcirculation occur irrespective of the systemic hemodynamic changes, although in the latter case this conclusion is disputed [14]. In the case of hypovolemic shock (caused, e.g., by a significant blood loss, and accompanied by pronounced hypotension), the changes in microcirculation are, at least, not completely independent from the hemodynamic parameters. The hemodynamic parameters are correlated with the “no-reflow” phenomenon [17, 18]. One of the definitions of the “no-reflow” phenomenon is incomplete and nonunified reperfusion at the microcirculatory level, despite an adequate reopening of the proximal artery after a period of transient ischemia [19]. The cases of vascular damage of the myocardium allow us to generalize the concept of no-reflow as the state of myocardial tissue hypoperfusion with the patency of epicardial coronary arteries [15, 18, 20]. Two types of no-reflow are distinguished: “structural” no-reflow, characterized by irreversible damage to the cells of the microcirculation, and “functional” no-reflow, characterized by the disturbance of microcirculation caused by vasoconstriction and/or microembolization [21]. The latter can be caused by the fragments of a lysed thrombus, destroyed atherosclerotic plaque, aggregates of erythrocytes or leukocytes with platelets, or by large platelets [15, 20, 22]. The high volumetric content of necrotic material in the plaque correlates with the development of microembolization, determined by the levels of the released seromarkers—creatine kinase and troponin I [23]. The stenting causes less prominent release of cardiac markers than does the atheroablative technology. These interventions can be associated with “reperfusion” no-reflow (which occurs after ischemia/reperfusion during the treatment of AMI) and with “interventional” no-reflow (associated with angioplasty without myocardial infarction and without a prolonged ischemia prior to the procedure) [15]. The duration of ischemia can have a significant effect on specific manifestations of no-reflow. “Interventional” no-reflow following a short period of ischemia (seconds or minutes) is caused primarily by microvascular obstruction, inflammatory response, or secondary ischemia. A prolonged (hours) ischemia causes no-reflow due to “reperfusion” (ischemia/reperfusion) injury, myocardial edema, endothelial swelling, capillary embolism, vasospasm, or inflammatory response [15]. The first 2-3 hours of ischemia in the case of AMI lead to myofibrillar edema, followed (6-7 hours of ischemia) by interstitial edema [24]. A more prolonged ischemia leads to the reduction of edema, to the destruction of microcirculation bed, and to the thinning of the myocardial walls. The inhibition of *δ*-isoform of protein kinase C by a fragment of its inhibitor, protein *δ*V1-1, performed on isolated hearts of transgenic mice expressing *δ*V1-1 and on AMI model in pigs reduces the microcirculatory dysfunction, decreasing the no-reflow phenomenon [25, 26]. The elevation in the levels of plasma thromboxane A2 was observed in the patients with AMI (with ST-segment elevation on ECG), following the primary stenting [20, 22]. The data of the multivariate analysis shows that the level of thromboxane A2 is an independent predictor of the no-reflow phenomenon after the angioplasty, which sets new goals for reducing of this complication. However, the efforts of pharmacologists and clinicians to achieve the same results using adenosine, sodium nitroprusside, nitroglycerine, nicorandil, verapamil, glycoprotein inhibitor IIb/IIIa, and papaverine have not as yet produced any tangible results [15, 26]. The multifactorial nature of the no-reflow development is emphasized by the assumption that the formation of fibrin clots with reduced permeability and increased resistance to fibrinolysis can be affected in these conditions by the genetic data of the patient [27]. The diversity of the available data and the lack of an effective approach to the therapy of no-reflow phenomenon make this goal the next challenge in treating the patients with AMI [20, 26]. The personalized management of no-reflow has been proposed for clinical application on the basis of the assessment of the prevailing mechanisms of no-reflow operating in each patient [28]. Variable combinations of human no-reflow causes include four pathogenetic components: distal atherothrombotic embolization, ischemic and/or reperfusion injury, susceptibility of coronary microcirculation to damage. In order to understand the mechanism of this pathology, we should know its components. The review presented above shows that the investigators tend to neglect the role of the carbohydrate coating of the microvessels, which (due to the reduction of the effective size of the vascular lumen and due to the vessel walls coming closer together and to the occlusion material) cannot be completely uninvolved in the development of the microcirculation disorders [29].

## 4. Carbohydrate Coating of the Vascular Wall

The endothelial glycocalyx (EG) is an uneven “fluffy” polysaccharide coating over the vascular wall toward the vessel lumen. This polysaccharide “fur” on the cell surface is composed of proteoglycans and glycoproteins, attached to the cell membrane and capable to bind blood constituents, which can be important for the vascular function. The size of the glycocalyx in the microvessels is 0.4-0.5 *μ*m, which is 10–20% of the vascular volume [30]. The diameter of the cell-free layer in these vessels is over 20 *μ*m [31]. It is believed that the glycocalyx permeability is mediated by the hyaluronan, and the glycocalyx volume is regulated by the proteoglycans [32, 33]. The composition of glycosaminoglycans implies the content of hyaluronan more than 40%, heparansulfate more than 50%, and chondroitinsulfate/dermatansulfate around 10% [34]. These glycosaminoglycans (besides hyaluronan) are linked covalently to core proteins [34–36]. They together with glycosaminoglycans form the proteoglycans. Core proteins are syndecans (number of subtypes is four) and glypicans (number of subtypes is six). Syndecans are linked firm to cell membrane via a membrane-spanning domain, but glypicans are linked via a glycosylphosphatidylinositol anchor. There are membrane proteoglycans (syndecans of subtypes 1–4 and glypicans of subtypes 1–6), soluble proteoglycans (perlecan, biglycan, and others), and five types of glycosaminoglycan chains (heparin sulfate, chondroitin sulfate, dermatan sulfate, keratan sulfate, hyaluronic acid). These components consist of membrane-bound and associated layers of glycocalyx. Composition and function of endothelial glycocalyx are detailed in [34, 36]. It should be noted that glycocalyx composition cannot be viewed as a static object. There is a dynamic equilibrium between glycocalyx layer of soluble components and the flowing blood. It is possible to say the glycocalyx is an intricate self-renewing 3D mesh of various polysaccharide derivatives [34–36]. EG is therefore considered to be a protective layer on the vascular wall providing defense against pathogens, a traffic meshwork barrier for transendothelium transportation of molecules, and a porous hydrodynamic partner in interaction with red and white blood cells in the microcirculation.

 The resistance of arterioles to the protein flux through their walls was found to be associated with the glycocalyx influence [37]. The inflammation and the influence of ischemia/reperfusion facilitate the breakdown of glycocalyx in the venules and capillaries [38–40]. When microthromboses in mice were induced by an infective stimulus (lipopolysaccharide), the levels of von Willebrand factor expression were increased in the venules, but not in the arterioles [41]. The degradation of glycocalyx in the myocardial capillaries results in a rapid development of myocardial tissue edema [42]. The treatment with hyaluronidase of hamster capillary glycocalyx has shown that it is the pronounced hyaluronandependent [30]. Hyaluronidase treatment was associated with the reduction of the glycocalyx volume, the increase of the volume of circulating plasma, the decrease of FCD, and the elevation of hematocrit. These changes in the glycocalyx lead to the alteration of the transendothelial permeability, thereby causing the swelling of the endothelial cells, which is one of the regulatory factors for FCD [30]. The reduction of FCD shows how the worsening of the condition of capillary glycocalyx can lead to the onset of the disease. The properties of the microcirculation glycocalyx can mediate the regulation of the blood transport function; these properties have recently been reviewed [29]. In humans, the evidence for shedding of EG during ischemia/reperfusion procedures was demonstrated already also [43].

## 5. Glycocalyx Components for Drug Targeting

The possibility of using glycocalyx components in the targeted drug delivery systems appears to be both interesting and promising [44]. The exposure of chondroitinsulfate proteoglycan in the subendothelial layer of the rabbit arteries after its stenting causes binding of the positively charged liposomes to this substance (along with cationic lipid TRX-20 and prednisolon serving as the drug) and the accumulation of such nanoparticles in the lesion area [45]. This has resulted in a prominent reduction of the neointima growth in the area of the stent. The treatment of the affected area with chondroitinase ABC prior to the injection of liposomes prevents their binding in this area. The experiment conducted on subendothelial cells (from human aorta) has confirmed a noticeable binding of the cationic TRX-20 liposomes to these cells, but not to the endothelial cells [46]. The treatment of subendothelial cells (smooth muscles and mesangial cells) with chondroitinase (but not with heparinase) reduces their binding to the liposomes studied, which proves the specificity of this interaction. It should be noted that liposomes can measurably bind to the endothelium, depending on the type of cationic lipids and their concentration in the liposomes [47]. The effects of binding of various ligands (lipoproteins, proteins, peptides, saccharide derivatives, etc.) to the components of EG have recently been reviewed [29]. Erythrocyte-associated tissue-type plasminogen activator (tPA) was proved to have a potential of a very effective agent of cerebrovascular thromboprophylaxis [48]. Biotinilated tPA and erythrocytes are associated via streptavidin [49]. The use of this adduct in mice with cerebral thrombosis results in thrombolysis, generating a rapid and prolonged reperfusion, whereas no effect is observed when tPA itself is administered even in ten-fold higher doses. Because of the coupling with erythrocytes, tPA has a prolonged half-life period in the blood, the ability to lyse fresh thrombi (rather than old hemostatic plugs), and a reduced efficiency of inhibition by plasminogen activator inhibitor of type I (PAI-1) [49]. The latter observation was explained by the protection provided by the erythrocyte glycocalyx preventing tPA from interacting with PAI-1, which disappears when the erythrocytes are treated with a mixture of neuraminidase, hyaluronidase, and heparinase [50]. The erythrocyte glycocalyx does not prevent the associated tPA from interacting with fibrin and plasminogen, but protects it from glycation by glucose. Of course, the similarity and difference of glycocalyx on leucocytes, erythrocytes, subendothelial cells to that on the endothelium has to determine else. A protective effect of glycocalyx is thought to be associated with screening the interaction centers on tPA provided by glycocalyx and with changes in electrostatic interactions. In our opinion, such effects (binding of the cationic liposomes to chondroitinsulfate-, but not to heparansulfate-proteoglycan [45], inhibition of the associated tPA interaction with PAI-1, but not with fibrin and plasminogen [49], that occur in the space of specifically positioned electrostatic charges) imply the presence of a suitable charge distribution network in glycocalyx. The orienting effect of this network on the binding counter-partner enables the detection of its suitability/unsuitability for a productive interaction. The presence of this charge distribution network can signify a specific ultrastructure of the glycocalyx. The study of this network is currently underway [29, 50].

## 6. The Study of Glycocalyx Ultrastructure

**Figure 1:**
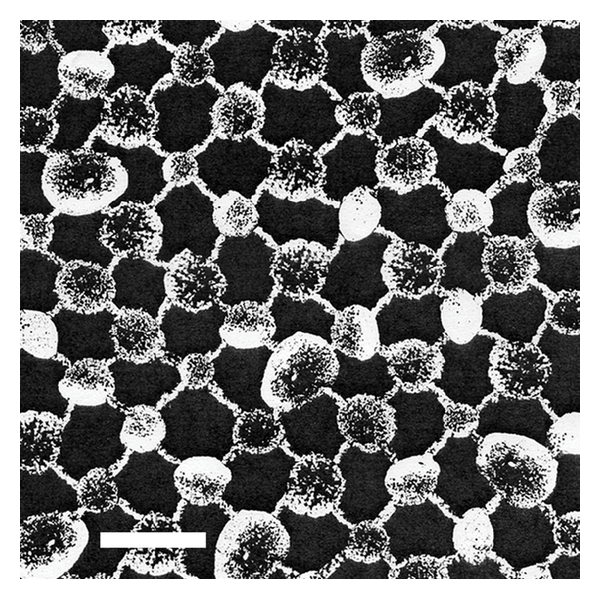
Reconstructed high-periodicity meshwork of the blood leukocyte glycocalyx composed of individual natural nanoparticles. The meshwork composition is projected onto a plane. Electron microscopy: the bar corresponds to the minimum size of the nanoparticle, which is 50 nm under 15% NaCl electrolyte concentration (hypertonic solution) or 3 nm under 0.9% NaCl electrolyte concentration (isotonic solution) (Golovanov MV, Bauer J, unpublished data personal report, 2009).

**Figure 2:**
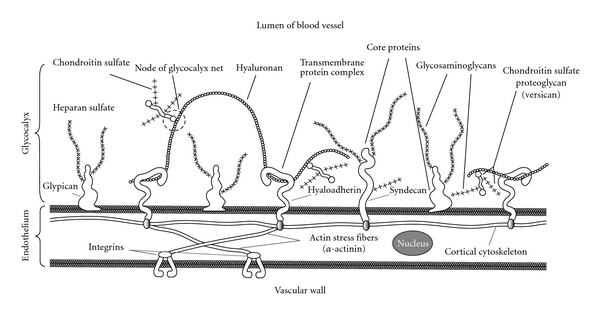
Schematic representation of the glycocalyx meshwork associated with cytoskeleton. Hyaluronan weaves into glycocalyx and binds with hyaladherins (CD-44 (hyalreceptor), RHAMM (receptor for hyaluronan-mediated motility), other proteins, or hyaluronansynthases). Chondroitin sulfate proteoglycan (versican) interacts with hyaluronan to form high molecular mass stable aggregates. Enhanced formation of hyaluronan-versican pericellular coat is observed under pathophysiological conditions (inflammation, early atherosclerosis, restenosis, plaque thrombosis, and others).

The samples of microvascular (capillaries and venules) glycocalyx from the frog endothelium prepared for electron microscopy using various techniques (freezing and chemical fixation) have yielded similar results [51]. The size of glycocalyx in the normal microvessels was less than 0.2 *μ*m; it was, however, observed that its *in vivo* size could be much higher and could be reduced, when the samples are prepared for electron microscopy. In the inflamed microvessels, where inflammation was induced using “temperature jump” technique, the glycocalyx changed its shape to form protuberances on the cell surface (extensions, depressions, and other irregularities) and increased in thickness (0.3-0.4 *μ*m). Computerized autocorrection functions and Fourier transformations reveal the ultrastructure of EG in the microvessels. A structural quasiperiodicity of the glycocalyx meshwork was detected along the horizontal and vertical axes (the periodicity intervals are ~20 nm, the diameter of fibers is 10–12 nm, and the distance between the bases of quasihexagonal meshwork elements (Figure 1) is approximately 100 nm [51]). This structure is consistent with the fiber matrix model that views the glycocalyx as an extracellular molecular filter. A systematic variation in the length of the side proteoglycan chains on the cell surface (axial periodicity along the proteoglycan molecule), and/or regulatory binding of plasma proteins (e.g., such ones albumin and orosomucoid [36]) by highly charged side chains of glycoproteins regularly positioned in the nodes of the glycocalyx network can serve as regulatory forces that mediate the transport through the glycocalyx (Figure 2) [51]. A specific profile of the electrostatic surface potential formed by this network is likely to trigger the initiation of recognition/binding of the interaction counter-partner (with a sufficiently complementary electrostatic surface potential) or to ignore the unsuitable counter-partner. This could have explained a targeted binding of the cationic liposomes to chondroitinsulfate-, but not to heparansulfate-proteoglycan [45, 46], as well as the inhibition by the erythrocyte glycocalyx of tPA interaction with PAI-1, but not with fibrin and plasminogen [49, 50]. This question definitely warrants further study.

 The treatment of the blood cells, intended for scanning electron microscopy, with 2.56 M (15%) NaCl solution [52] provides a very informative approach for studying this problem. During this procedure, gel microenvironment is formed around the cells (taken from the patient blood with chronic lymphatic leukemia or myeloleukemia); this microenvironment has a regular meshwork structure. The cell diameter, including the swollen glycocalyx-gel around the cell, is approximately 10–20 *μ*m. The meshwork periodic structure (period of 100–150 nm) of the tumor cells was clearly revealed by atomic force microscopy [53]. A reversible increase in size of the cellular glycocalyx reaches three orders of magnitude [52, 53], which makes this approach a very convenient technique for studying the structure of glycocalyx.

 At the same time, it is assumed [50] that the organization of the glycocalyx ultrastructure (100 nm topographic distances between the glycocalyx fibers on the cellular surface, Figure 1) is determined by the quasiregular architecture of the submembrane cytoskeleton. It is possible that the inflammation alters the glycocalyx by changing the structure of the cytoskeleton, which in turn triggers the over-production of the glycocalyx [51]. This change can be initiated as a result of the effect that the high ionic strength of the medium has on the cytoskeleton of the tumor cells and/or as a result of the increased swelling of the glycocalyx [52, 53]. The quantitative study of the proposed structural glycocalyx model [51] has shown that a high flexural rigidity of the fibers on the cell surface is provided by the support of the rigid actin filaments of the cortical cytoskeleton in the transmembrane complexes of the core glycocalyx proteins [54]. As a result, the submembrane “roots” of the glycocalyx “bushes” are holding firmly the “branches” of their fiber clusters (Figure 2). The long arm of the lever formed by the core glycoproteins on the outside of the cell provides significant mechanical advantages inside the cell in terms of increasing the flux forces, exerted on the glycocalyx, in transferring the interaction to the cortical cytoskeleton. Small flux forces acting on the glycocalyx are amplified by a sufficient intracellular lever, capable of deforming the cortical cytoskeleton. It is assumed that such mechanotransduction is the first step in the activation chain of intracellular signaling [54]. Hyaluronan, a component of the glycocalyx, can interact with the cytoskeleton through CD44 and RHAMM, the hyaladherins associated with the glycocalyx [55]. RHAMM is associated with the microtubules (of the mitotic cells), around which the internalized hyaluronan is distributed [56, 57]. The microtubules are an element in the development of the cytoskeleton pressure for regulating cellular junctions and cell shapes [58]. In this connection, it should be mentioned that the appearance of cilia and flagella on the endothelial surface, formed by the centrosomal centrioles and assembled from the microtubules, results in the development of endothelial dysfunction in the areas of high-risk circulation (with an oscillating shear stress) [59]. Moreover, endothelial cilia can serve as regulators of the calcium signaling and NO production via polycystin-1 [60, 61]. Enzyme destruction of EG (treatment with heparinase, hyaluronidase, neuraminidase, excluding chondroitinase) completely blocked shear-induced NO production by endothelial cells [62, 63]. None of these enzymes affected bradykinin- or histamine-induced NO production, indicating the multeity of NO production machinery. Meanwhile, it conjectures the glycocalyx may be organized into two layers: an inner region of several tens of nm near the apical membrane surface and an outer layer up to 0.5 *μ*m thick, which contains the extended core proteins [64]. Between these layers may be located hyaluronan. Heparansulfate seems to locate in outer layer due to its prevailing content among glycocalyx glycosaminoglycans [34] and observable role in mechanotransduction of shear stress into endothelial cells for NO production (Figure 2) [65]. Under physiological conditions, EG has several well defined functions aimed at preserving the integrity of the vessel wall: inert barrier, molecular sieve, reservoir for biologically active compounds, mechanotransductor transferring shear stress into shear-dependent endothelial responses [64]. As a whole, according to the “double barrier concept,” vascular barrier function is provided by two important components, the endothelial glycocalyx and the endothelial cells themselves [40, 43]. It can be hypothesized that extracellular stimuli are conveyed to the cytoskeleton through the structure of the glycocalyx, and then the endothelial cell uses the cytoskeleton to develop a response, which can result, for instance, in a change of cell shape or an initiation of new events that alter the cellular functions.

## 7. Glycocalyx Degradation and Its Fragments in Blood Circulation

The degradation of EG due to the development of vascular lesions leads to the release of its fragments into the circulation. These fragments are currently believed to be glycosaminoglycans, previously attached to the proteins on the endothelial surface. The molecular size of hyaluronan far exceeds the sizes of other glycocalyx glycosaminoglycans. The hyaluronan molecule can be as long as 2.0–2.5 *μ*m [55]. The effects associated with the formation of the hyaluronan fragments have already been discussed in the literature [55, 66, 67]. There is a mounting body of data showing different types of biological activity of the hyaluronan fragments, depending on their molecular weight [29]. The full-size forms of hyaluronan have proved to have anti-inflammatory and anti-angiogenic properties, the oligomers of 4–50 disaccharides have angiogenic and pro-inflammatory properties and stimulate tumor invasions, whereas the fragments of 3–12 and 6-7 hyalurononic polymeric moieties were found to suppress tumor growth [66, 67]. The degradation of hyaluronan is important for the functioning of the capillaries because hyaluronan regulates their permeability and the junctions between endothelial cells [30, 68]. Hyaluronan fragments are released in the circulation due to biochemical degradation of the glycocalyx, *de novo* synthesis, and the effect of the oxidative stress [29, 67]. Inflammatory processes caused by the oxidative fragmentation of hyaluronan, were inhibited by the extracellular superoxide dismutase [69]. The exogenous hyaluronan fragments, from decasaccharides upward, displace hyaluronan from the cell surface, whereas the chondroitinsulfate does not have such effect [70]. The splitting of CD44 was caused by the small fragments of hyaluronan (6.9 kDa and other fragments with molecular weight less than 36 kDa), which in turn led to the increase of tumor cell mobility due to detachment/dissociation of these cells from the tumor [71]. Hyaluronan oligosaccharides (composed of 4–16 saccharides) inhibit neointima growth in the area of the rat aorta affected by balloon catheterization [72]. This makes such hyaluronan fragments potential agents for preventing restenoses following the angioplasty, because their formation is caused predominantly by the growth of the extracellular matrix, rather than by cell proliferation [55, 73]. Hyaluronic acid was proposed to be used as thromboresistant coating of the stents and endovascular devices [74].

 Heparin, known for its anticoagulative properties, can interact with fibronectin, a protein of the extracellular matrix, thereby triggering the transformation of its molecule on the cell surface from closed to open conformation [75]. The binding of heparin to the open conformation is weakened, heparin dissociates from the protein, and the binding centers of vascular endothelium growth factor become exposed on the open conformation. Heparin, for instance, can regulate the functions of extracellular matrix.

 The enzymatic destruction of glycocalyx is accomplished by neuraminidase, chondroitinase, heparinase, hyaluronidase, and other biocatalysts [26, 46, 50, 55, 67]. The treatment with mammal hyaluronidase results in the formation of oligosaccharides with an even number of polymeric moieties that have *N*-acetylglucosamine at the reducing end of the fragment [67, 76]. Such derivatives give rise to glycating agents [77]. The glycation of biomacromolecules, especially in carbohydrate disorders, results in formation of Amadori end-products, whose accumulation in the body tissues facilitates the development of metabolic disorders, the increase in rigidity of the vascular wall, and production of the reactive oxygen species [77–80]. The interaction of Amadori end-products (advanced glycation end-products) with their corresponding receptors can facilitate these processes, by affecting the energetics of myocardial metabolism, its function, and by contributing to the myocardial damage after ischemia/reperfusion [81]. In order to achieve therapeutic effect, the formation of Amadori end-products should be blocked and a soluble form of the receptor should be used [82].

 The glycosaminoglycan biosynthesis comprises several steps. The glycosaminoglycan chain initiation is catalyzed by xylosyltransferase-1/2 that transfers xylose residues to a certain serine in the core protein. This is the first step in the assembly of the linkage tetrasaccharide GlcA*β*(1–3)Gal*β*(1–3)Gal*β*(1–4)Xyl*β*(1-O-Ser) linking the protein and the future glycosaminoglycan chain. Galactosyl transferase-1 and -2, and glucuronyl transferase-1 complete the assembly of the linkage tetrasaccharide by a consecutive transfer of two galactose residues and one residue of glucuronic acid. The subsequent elongation of the chain is accomplished by an alternate addition of the transferred units of D-glucuronic acid and *N*-acetyl-D-glucosamine to the tetrasaccharide. The final step in the synthesis of proteoglycans is associated with highly coordinated multiple sulfation and epimerization of the glycosaminoglycan. The biological significance of glycosaminoglycans is studied by exposing them to various agents in the course of the glycosaminoglycan biosynthesis. The following substances can serve as agents that change the glycosaminoglycan biosynthesis: sodium chlorate, a bleaching agent, and brefedlin A, a fungal metabolite. Unfortunately, these agents turned out to be highly lethal for the animal models. The modified analogues of xylose were proved to be more suitable for studying [83]. These studies of xylose analogues are currently underway and they can help to examine the biological role of glycosaminoglycan derivates, synthesized *de novo *in the organism.

## 8. Present Methods for Glycocalyx Study

In order to evaluate glycocalyx state, various methods employing reporter agents (isotopes, labeled erythrocytes, dextran) and novel research equipment (laser doppler flowmetry, positron emission, single-photon computerized, magnetic resonance (using gadolinium, iron oxide) tomography or their combinations) are used [14, 84, 85]. There are ongoing studies looking for correlations between the endothelial glycocalyx thickness in the microcirculation and the risk factors for cardiovascular disorders. The novel microcirculation imaging methods that proved effective during the studies with human volunteers are now being developed: orthogonal polarization spectral imaging (OPS, measured in the sublingual area) [86] and sidestream dark field imaging (SDF, measured on the nail fold) [14]. Cardiovascular magnetic resonance imaging of myocardial edema detects acute ischemic myocyte injury before the onset of irreversible damage [87]. This imaging may be a useful diagnostic marker in clinical settings (unstable angina or evolving infarction). The integration of the imaging techniques with glycocalyx degradation products in plasma will conduce in elicitation of EG role for cardiovascular risk stratification. EG is present in macro- as well as microvasculature and its thickness progresses with increasing vascular diameter [34]. In larger vessel, the two-photon laser scanning microscopy is suitable technique to visualize the delicate EG [88, 89]. The glycocalyx is thought to be a similarity in capillaries and large vessel throughout the whole body [42]. It is not strange therefore that arterial glycocalyx dysfunction could consider the first step in atherothrombosis process [90]. Glycocalyx is implicated also in development of diabetes, atherosclerosis, and ischemia/reperfusion injury [36]. The predicting the future is ungrateful job but further research of EG looks like the hopeful and breakthrough investigation in order to decrease the spread of cardiovascular diseases.

## 9. Conclusion and Perspective

The progress in reperfusion therapy leads to the necessity of developing new tools for adjacent treatment of cardiovascular disorders. One of the purposes of this treatment is the damages of the microcirculation in acute coronary syndrome. The unfavorable prognosis for the patients without “optimal” reperfusion places them in the high risk groups that have the no-reflow phenomenon in microcirculation. The clinical significance of successful treatment of this disorder offers a new challenge in treating the patients with AMI associated with ST-segment elevation. The studies of the no-reflow phenomenon reveal various causes for its development, paying, however, clearly insufficient attention to the role of EG. The study of the glycocalyx demonstrates that this spatial meshwork structure provides a protective function for the cell surface and that glycocalyx is capable to serve as a molecular filter and hydrodynamic partner in the interaction with cells and blood components. The action of chemical (enzymes, reactive oxygen species, changes of the medium pH value) and physical (shear stress, temperature, ultrasound, photo- and radio- emission, etc.) factors changes the structure of the glycocalyx, which is currently intensively studied and can influence the extent of the tissue perfusion. A diverse biological activity of EG fragments is also clearly evident and can substantially differ, depending on the molecular weight of these fragments. It is assumed that the glycocalyx, being connected to the cytoskeleton, serves as a mechanochemical transducer of the effect that blood circulation has on the processes of cell signaling. This may suggest a predictive role for the state of glycocalyx in the microcirculation. The path leading to our understanding of the mechanism for these effects is a very difficult way. However, an intention of following this path is justified by the prospective of successful treatment in the patients with complicated cardiovascular disorders.

## Abbreviations

AMI: Acute myocardial infarction
CD44: Hyalreceptor
ECG: Electrocardiogram
EG: Endothelial glycocalyx
FCD: Functional capillary density
OPS: Orthogonal polarization spectral imaging
PAI-1: Plasminogen activator inhibitor of type 1
RHAMM: Receptor for hyaluronan-mediated motility
SDF: Sidestream dark field imaging
tPA: Tissue-type plasminogen activator.
